# Eosinophilic enterocolitis: a case report

**DOI:** 10.1186/s13256-023-04319-9

**Published:** 2024-01-19

**Authors:** Hend Smaoui, Abdelwaheb Nakhli, Nesrine Hemdani, Bochra Bouchabou, Rym Ennaifer

**Affiliations:** Mongi Slim University Hospital, Tunis, Tunisia

**Keywords:** Eosinophilic colitis, Acute bowel obstruction, Extensive ileitis, Steroids

## Abstract

**Background:**

Eosinophilic enterocolitis is a rare disorder characterized by abnormal eosinophilic infiltration of the small intestine and the colon.

**Case presentation:**

We report a case of a 29-year-old White man, who presented with an acute bowel obstruction. He had a history of a 2 months non-bloody diarrhea. An abdominal computed tomography (CT) and a MR enterography showed a multifocal extensive ileitis. White blood cell and eosinophilic polynuclei count was elevated (700/mm^3^). Ileo-colonoscopy showed normal ileum and segmental petechial colitis. Pathology showed a high eosinophilic infiltration in the colon. The patient was treated with steroids, with a clinical, biological and radiological recovery.

**Conclusion:**

Eosinophilic enterocolitis should be kept in mind as a rare differential diagnosis in patients presenting with small bowel obstruction.

## Background

Eosinophilic enterocolitis (EC) is a rare condition included in the group of eosinophilic gastrointestinal disorders (EGIDs). It is characterized by a high eosinophilic infiltration in the gut wall [1]. EC may be primary, without a known etiology, or secondary to an identified cause [2]. There is a slight female and Caucasian preponderance. The clinical presentation is variable and symptoms of abdominal pain, weight loss, diarrhea, bloody stools, and malabsorption are described. When the whole bowel wall is involved, intestinal obstruction and even perforation may be seen [3]. Peripheral eosinophilia is inconstant. The definitive diagnosis is made on biopsy [4].

## Case presentation

A 29-year-old smoking White man, without any personal or family history, was hospitalized in our department for the management of a small bowel obstruction (SBO). There was no fever or night sweats. The general condition was preserved. He had a history of a 2 months non-bloody diarrhea (5 stools/day). At presentation, physical examination revealed marked abdominal distension, diffuse tympanism with tenderness without rebound tenderness. There was no fever and vital signs were stable. Neurological and cutaneous examinations were normal. Examination of the anal margin and the rectal examination were normal.

Abdominal CT scan revealed segmental, multifocal thickened small intestinal walls (8 mm) and dilated loops in the small bowel (up to 41 mm). The thickening was circumferential with a target appearance due to submucosal oedema.

White blood cell and eosinophilic polynuclei count was elevated (700/mm^3^). Hemoglobin value was 12.8 g/dl and platelet count was within normal ranges. The C-reactive protein value was elevated (96 µmol/l). Liver and kidney function tests were normal. The SBO had improved with conservative management.

Parasitological examination of stool and stool culture were negative. Quantiferon, ASCA, PANCA and anti-transglutaminase antibodies were negative. LDH levels were normal.

**Fig. 1:**
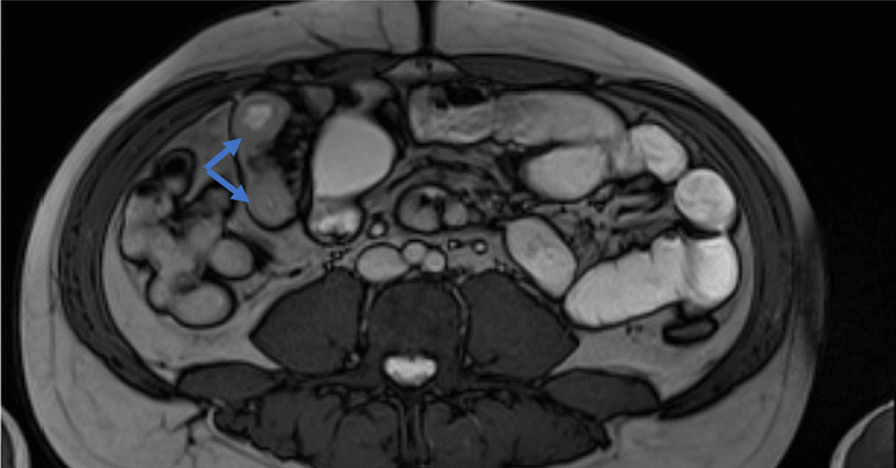
MR enterography showing a discontinuous an inflammatory thickening of the ileum (Blue arrows indicate an inflammatory thickening of the ileum)

**Fig. 2:**
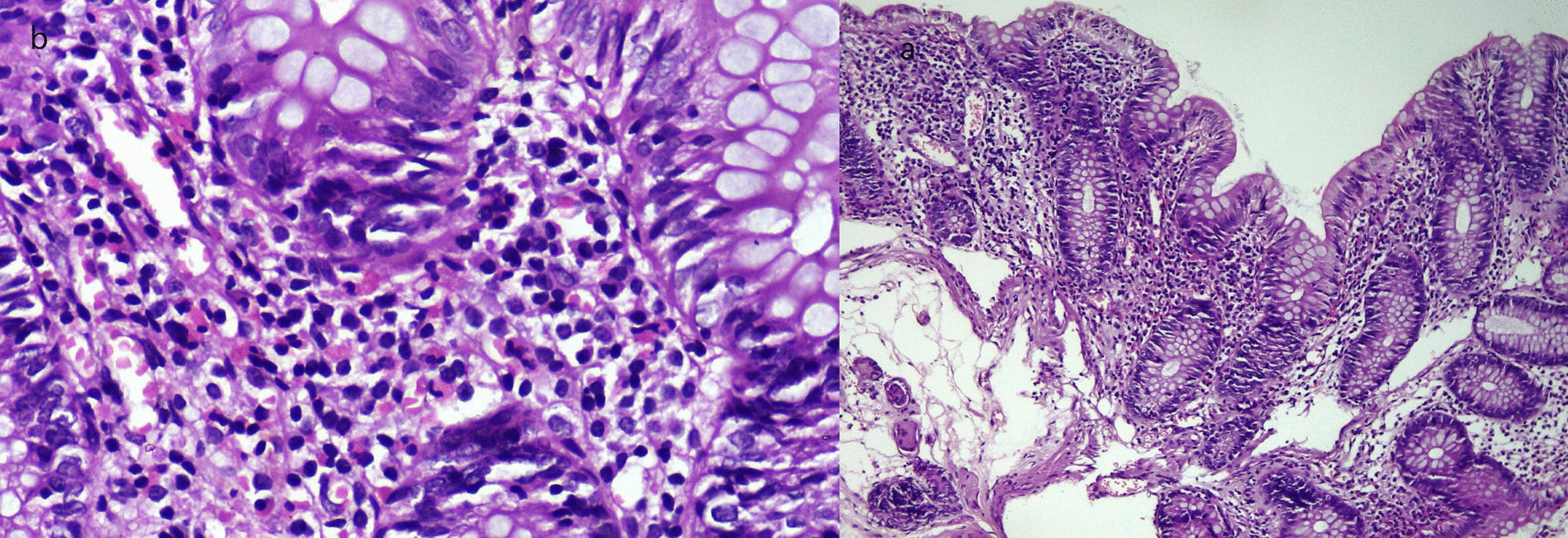
**a** Low magnification image showing an increased cellular density in the chorion without cryptic dystrophy or signs of chronicity; **b** image showing more than 60 eosinophilic granulocytes per field at high magnification

The MR enterography showed a discontinuous multifocal inflammatory thickening of the ileum (Fig. 1). Ileo-colonoscopy showed normal ileum and segmental petechial colitis. Pathology was normal for ileal biopsies and showed a catarrhal0 colitis with high eosinophilic infiltration without epithelial architectural changes for colonic biopsies (Fig. 2). The gastroscopy showed a congestive and petechial gastropathy. Pathology was normal for esophageal and duodenal biopsies and showed chronic gastritis without HP for gastric biopsies.

The patient did not have antibiotics, since the Parasitological examination of stool and stool culture were negative. He was diagnosed with primary eosinophilic enterocolitis. He received corticosteroid therapy. We observed the resolution of the subocclusives syndromes, the diarrhea and the biological inflammatory syndrome, the normalization of the PNE level. Control MR enterography was normal three months after corticosteroid therapy. Since the patient was asymptomatic, we did not do a second look endoscopy.

After a year, the patient was asymptomatic and the biological tests were normal.

## Discussion

EGID is an uncommon, chronic condition of the digestive tract, characterized by eosinophilic infiltration of the gastrointestinal wall, typically involving the stomach, small intestines, and, less commonly, the colon [5]. Peripheral eosinophilia is inconstant. EC is the rarest reported form of EGID, with only few reported cases in adults, although there has been a rise over the last decade. In a large-scale retrospective epidemiological study conducted in the United States, the incidence of EC was 3.7 per 100,000 population, and that of primary EC was 2.4 per 100,000 population [6, 7]. It most often occurs between the third and fifth decades of life.

EC can be classified as primary or secondary [2]. Secondary EC results from either an eosinophilic disorder, such as a hypereosinophilic syndrome, or pathologies unrelated to an eosinophilic disorder, such as inflammatory bowel diseases, parasitic infections, certain drugs and systemic diseases (Table 1). In the majority of cases, primary EC are related to an allergic reaction, either IgE-mediated and at the origin of an anaphylactic type of food allergy, or not mediated by IgE and at the origin of food enteropathy, with milk proteins being the main food involved in children’s EC [8]. The most common allergic diseases associated with EC are rhinitis, eczema and asthma [7].

**Table 1:** Etiologies of secondary eosinophilic colitis

Eosinophilic colitis not related to a eosinophilic disorder	Parasitosis	Low: Oxyurosis, Taeniasis, Trichocephalosis, Anguillulosis, Angiostrongylosis. High: Trichinosis, Toxocarosis, bilharziasis, Ascaridiosis, Ankylostomosis
	Drugs	Clozapine, carbamazepine, rifampicine, tacrolimus, gabapentine, pregabalin, sulphasalazine, non-steroidal anti-inflammatory, gold salts
	Chronic inflammatory bowel diseases Ulcerative recto-colitis Crohn’s disease	Ulcerative recto-colitis Crohn’s disease
	Autoimmune diseases	Scleroderma, Churg-Strauss syndrome, celiac disease, dermatitis herpetiformis, systemic lupus erythematosus, pemphigus, periarteritis nodosa, rheumatoid arthritis, psoriasis, sarcoidosis, Sjögren’s syndrome
	Blood diseases Solid neoplasia	Hodgkin’s or non-Hodgkin’s lymphoma, some leukemias. Breast, liver, kidney and thyroid cancer
	Allogeneic marrow transplantation Syndrome of Tolosa-Hunt	Headache, ophthalmoplegia, paralysis of cranial nerves
Eosinophilic colitis related to eosinophilic disorder Hyper-eosinophilic syndrome		Persistent elevation of eosinophilia (≥ 1500/mm^3^) for at least 6 months. No cause of secondary eosinophilia

Symptoms of EC are not disease-specific [9]. Diarrhea is the most frequent symptom, present in more than 60% of cases, while rectal bleeding is only found in 10 to 20% of the cases studied. Abdominal pains are also common, being observed in 60 to more than 80% of cases. Nausea and vomiting were noted in about 30% of cases. A minimal loss of weight is also possible [7].

The presentation of EC tends to depend on which intestinal layer is most affected by the eosinophilic infiltration. In 1970, Klein *et al.* divided these diseases into three types depending on the depth of eosinophilic infiltration (Table 2) [10].

**Table 2:** The classification described by Klein *et al.* [10]

Mucosal	• The most common • Non-specific symptoms of abdominal pain, nausea, diarrhea, and vomiting ± more severe symptoms related to blood loss in stools blood loss in stools, iron deficiency anemia, malabsorption, or a protein-losing enteropathy
Muscular	*•* Infiltration of eosinophils predominantly in the muscle layer, causing bowel wall thickening and, in turn, symptoms of intestinal obstruction
Serosal	*•* The least reported form *•* Isolated abdominal ascites or ascites along with symptoms

EC may also present as perianal disease [11], chronic intestinal pseudo-obstruction [12], and appendicitis [13].

Laboratory tests are of limited value due to their low sensitivity and specificity. The blood eosinophilia is a good biological marker but is not constant and is sometimes transient [14]. In adults, the increase in serum total IgE levels is also inconstant and the search for IgE specific to certain foods is almost always negative. Atopy prick-tests and patch-tests are not recommended for diagnosing food allergies due to their limited diagnostic value.

Non-specific endoscopic findings, such as patchy areas of mucosal edema, punctate erythema, elevated lesions, pale granular mucosa and aphthous ulceration, may be seen, although these findings are uncommon and in most cases the mucosa is endoscopically normal [15]. There is no consensus concerning the physiological levels of eosinophils in the colonic mucosa [2]. Some authors have suggested that an eosinophil level of more than 40 per HPF in at least 2 different colonic segments is necessary to confirm the diagnosis of EC [2].

Management of EC is based essentially on case series and expert opinion [3].

The uses of elimination diets have been shown to improve clinical symptoms and reduce mucosal eosinophils but its efficacy depends mainly on patient compliance [16]. Symptomatic and histologic remission has been described with exclusively elemental diets and may be used as a steroid-sparing option [17]. Corticosteroid therapy (0.5–1 mg/kg/d tapered over 2–4 weeks) are considered first line pharmacological treatment [3]. Budesonide is an alternative that has also been shown to be effective with fewer side effects [18].

Multiple studies have reported efficacy and safety of Ketotifen—a histamine H1 receptor antagonist- and have proposed it as an alternative to corticosteroids [19]. The role of Montelukast, a selective leukotriene D4 receptor antagonist is still debated [20]. Finally, as there are similarities in the pathogenesis of EC with eosinophilic esophagitis (EoE), biologic medications undergoing clinical trials for the treatment of EoE are potential therapeutic agents for EC (Anti-IL5, anti-IgE monoclonal antibodies, anti-tumor necrosis factor (TNF-α) [4].

## Conclusion

Eosinophilic enterocolitis should be considered as a rare differential diagnosis in patients presenting with small bowel obstruction.

## Data Availability

Data and materials are available.

## Abbreviations

EC: Eosinophilic colitis
EGIDs: Eosinophilic gastrointestinal disorders
SBO: Small bowel obstruction
CT scan: Computed tomography scan
PNE: Eosinophilic polynuclei
EoE: Eosinophilic esophagitis
TNF-α: Tumor necrosis factor α
